# Cannabis use in first episode psychosis: what we have tried and why it hasn’t worked

**DOI:** 10.1186/s12916-019-1421-7

**Published:** 2019-10-28

**Authors:** Michael G. McDonell, Oladunni Oluwoye

**Affiliations:** 1 Elson S. Floyd College of Medicine, Washington State University, 412 E. Spokane Falls Blvd, Spokane, WA 99210-1495 USA; 2 Program of Excellence in Addictions Research, Washington State University, Spokane, WA USA

**Keywords:** Cannabis, First episode psychosis, Contingency management, Early intervention, Substance use, Co-occurring disorders

## Background

Up to 64% of individuals who have experienced a first episode of psychosis (FEP) have used cannabis, and 30% of these have a cannabis use disorder [1, 2]. Cannabis use among those with FEP is associated with negative outcomes (e.g., positive symptoms, illness severity) [1]. Given the relationship between cannabis use and psychosis, and the increasing availability of high potency tetrahydrocannabinol (THC) products, cannabis use prevention and treatment in people with FEP is a critical issue. Initial early intervention efforts that address cannabis and other substance use has been underwhelming. Early intervention programs for FEP generally include evidence-based practices focused on medication management, family psychoeducation, case management, individual therapy, and vocational services [3]. Brief or optional cognitive behavioral therapy for substance use is often integrated in early intervention programs. The question is, are these interventions sufficient in their intensity and/or duration to reduce cannabis use?

The answer appears to be no. Young people enrolled in early intervention programs do not have lower levels of cannabis or other drug use than those who receive treatment as usual [4]. In a recent secondary data analysis, we found that frequent cannabis use (> 20 days per month) was higher in those randomized to the early intervention treatment than those in usual care [5]. These findings are consistent with the treatment outcomes of general co-occurring disorders. Few interventions for co-occurring disorders are designed to treat both schizophrenia spectrum and substance use disorders. Most are focused on improving psychiatric symptoms (e.g., assertive community treatment) or increasing drug or alcohol abstinence (e.g., contingency management). As a result, few interventions including contingency management (CM) consistently demonstrate reductions in both psychiatric symptoms and substance use [6]. Although the addition of CM or other evidence-based interventions to early intervention programs for FEP is appealing, truly integrative treatments are needed to improve the lives of young people living with psychosis and substance use disorders.

## Is contingency management (CM) a viable treatment option for early intervention programs?

CM is an evidence-based intervention in which individuals receive tangible rewards for submitting objective evidence of drug and alcohol abstinence [7]. Our group observed significantly lower levels of inpatient psychiatric care and psychiatric symptoms in adult patients with serious mental illness, including schizophrenia, who received CM for stimulant drug abstinence [8]. We found the CM intervention to be cost-effective, in part because of reductions in inpatient utilization [9]. The CIRCLE trial, by Rains et al. [10], was conducted to determine whether or not CM reduced cannabis use and psychiatric hospitalization, and whether it was cost-effective compared to a psychoeducational intervention for cannabis use in an early intervention program for FEP. Overall, the findings equivocally supported positive clinical outcomes that could be attributed to CM. Interpretation of these results leads us to explore the development of CM or other approaches as treatments for cannabis use in FEP programs.

## Future directions

Findings from the CIRCLE trial raise several considerations for future research. First, for CM to be effective, the magnitude and frequency of rewards for abstinence must be sufficient to decrease use. The unique developmental and psychiatric profiles of those with FEP mean that typical CM schedules might require modification to improve the intervention efficacy (e.g., more frequent or higher magnitude rewards). Second, a conclusive trial of CM for cannabis or other substance use requires the combination of self-report and a biochemically confirmed outcome in both the CM and control groups. Third, while we have observed CM-associated reductions in psychiatric hospitalizations, our work investigated treatment as usual in public mental health centers in the USA. The CIRCLE trial, however, investigated the impact of CM on hospitalizations within the context of specialized FEP treatment, which is designed to reduce acute psychiatric care utilization [10]. Thus, it may have been challenging for CM to reduce acute care, above and beyond that of typical FEP treatment. Fourth, the inclusion of a psychoeducational intervention focused on cannabis use in the control condition may have had a positive impact on cannabis use, since use in both groups declined over time. This intervention might be an effective treatment for cannabis use, and warrants further study. Finally, the CIRCLE trial raises important questions about the feasibility of CM for FEP programs; the authors describe the challenges of adding a simple intervention, such as CM, to an already intensive, multi-component treatment for FEP [10]. Additional work investigating how CM and other substance use treatments can be implemented in FEP care is greatly needed.

## Conclusions

In the past decade, early intervention programs for FEP have revolutionized the treatment of schizophrenia. This momentum provides the opportunity to integrate interventions that address cannabis and substance use problems early in the course of illness, with the goal of preventing the negative effects of substance use on people with psychosis. With the increasing availability of high potency THC products, and the global movement towards decriminalization, cannabis use presents a formidable and important area of focus for FEP treatment providers. Studies such as the CIRCLE trial indicate that developing an effective treatment for cannabis use in this population will not be easy. A one-size-fits-all approach is unlikely to be effective given the homogeneity of cannabis use (e.g., differences in frequency or motivations for use) in young people with FEP. Effective strategies are also needed to prevent people with FEP from using cannabis in the first place. Future research focusing on implementation and dissemination issues is needed to close the gap between research and practice improvement. As a field, we should strive to develop truly integrated early interventions for psychosis and substance use to help improve the lives of young people with, or at high risk of, these commonly co-occurring disorders.

## Data Availability

Not applicable.
